# A urinary Common Rejection Module (uCRM) score for non-invasive kidney transplant monitoring

**DOI:** 10.1371/journal.pone.0220052

**Published:** 2019-07-31

**Authors:** Tara K. Sigdel, Joshua Y. C. Yang, Oriol Bestard, Andrew Schroeder, Szu-Chuan Hsieh, Juliane M. Liberto, Izabella Damm, Anna C. M. Geraedts, Minnie M. Sarwal

**Affiliations:** 1 Division of Transplant Surgery, Department of Surgery, University of California San Francisco, San Francisco, California, United States of America; 2 Kidney Transplant Unit, Bellvitge University Hospital, UB, Barcelona, Spain; University of Toledo, UNITED STATES

## Abstract

A Common Rejection Module (CRM) consisting of 11 genes expressed in allograft biopsies was previously reported to serve as a biomarker for acute rejection (AR), correlate with the extent of graft injury, and predict future allograft damage. We investigated the use of this gene panel on the urine cell pellet of kidney transplant patients. Urinary cell sediments collected from patients with biopsy-confirmed acute rejection, borderline AR (bAR), BK virus nephropathy (BKVN), and stable kidney grafts with normal protocol biopsies (STA) were analyzed for expression of these 11 genes using quantitative polymerase chain reaction (qPCR). We assessed these 11 CRM genes for their abundance, autocorrelation, and individual expression levels. Expression of 10/11 genes were elevated in AR when compared to STA. Psmb9 and Cxcl10could classify AR versus STA as accurately as the 11-gene model (sensitivity = 93.6%, specificity = 97.6%). A uCRM score, based on the geometric mean of the expression levels, could distinguish AR from STA with high accuracy (AUC = 0.9886) and correlated specifically with histologic measures of tubulitis and interstitial inflammation rather than tubular atrophy, glomerulosclerosis, intimal proliferation, tubular vacuolization or acute glomerulitis. This urine gene expression-based score may enable the non-invasive and quantitative monitoring of AR.

## Introduction

Kidney transplantation (KTx) is the preferred modality for treatment of end-stage renal disease (ESRD) by any cause [1]. While this therapeutic approach has become a routine practice worldwide, significantly improving patient quality of life and survival [2], long-term kidney allograft outcomes have not improved as expected despite a better understanding of the immune biology of allograft rejection and the advent of novel and more potent immunosuppressive agents [3]. The major cause for persistent and poor graft survival is due to the inability to non-invasively quantify the burden of graft immune injury and predict acute rejection prior to substantive functional decline and histological injury. Indeed, while it is well known that KTx patients are continuously exposed to immune and nonimmune related injuries [4, 5], periodic KTx monitoring is dependent on insensitive surrogate markers of allograft dysfunction such as serum creatinine [6, 7] and sporadic KTx monitoring is based on protocol allograft biopsies to detect sub-clinical histological graft injury in the absence of perturbation of the serum creatinine [8]. Though assessment of graft dysfunction based only on the serum creatinine has sensitivity for non-specific, established, allograft damage, it has low specificity for diagnosis of acute rejection (AR), as a rise in the serum creatinine can be due to other reasons not directly related to allograft rejection, such as immunosuppressive (IS) drug-related nephrotoxicity, acute tubular necrosis, infection, and interstitial fibrosis and tubular atrophy (IFTA). Furthermore, while the use of surveillance biopsies has been postulated as the gold standard tool for diagnosing allograft lesions, this approach is costly, invasive, with procedure morbidity (risk of bleeding; procedure requiring sedation, particularly for pediatric KTx patients) [9], fraught with inter-operator read variabilities, and often poorly representative of focal histological injury. Therefore, the use of non-invasive biological markers that can accurately predict and quantify the burden of immune injury in the allograft would be a significant advance for precision KTx monitoring [10–12].

Interrogation of proteomic, RNA, and microRNA biomarkers in the urine of KTx patients has been demonstrated by our groups and others [13–17] to be an optimal biological fluid for serial monitoring of the kidney allograft because it is an ultrafiltrate of the kidney and mirrors the biological processes and inflammatory burden found in the kidney graft [18]. Despite a number of studies that have previously evaluated urine biomarkers as a non-invasive diagnostic approach for the analysis of AR in kidney transplantation, the exclusive focus on single biomarkers such as particular chemokines and receptors such as CXCR3, CXCL9, or CXCL10 [19–24] make it difficult to capture the molecular complexity and heterogeneity of AR across different KTx patients. Capturing this heterogeneity is essential to quantify the burden of injury in a manner usable for prospective monitoring of AR and recovery of graft injury after therapeutic intervention [25, 26].

In this study, we apply the knowledge gained from harnessing a Common Rejection Module (CRM) of 11 genes [27], originally developed using exhaustive meta-analysis of publicly available transplant tissue microarray datasets of biopsy samples from four different types of solid organs. The CRM genes in tissue (tCRM) were all over-expressed among AR patients, irrespective of the type of organ, differences in immunosuppression protocols, or differences in the platforms interrogating gene expression. A quantitative threshold determined by computational analysis of a combined gene-score (the tCRM score) accurately predicted the presence of AR by cross-validation of tissue gene expression signatures in 8 independent cohorts (n = 236 samples) of human kidney allograft biopsies [27]. The tCRM score was further validated by qPCR in a separate study on KTx biopsy samples as diagnostic of both AR and chronic allograft injury (CAI) with different gene-set thresholds [28]. Furthermore, this set of CRM genes was validated in an independent set of biopsied tissue from lung transplant patients with chronic lung allograft dysfunction (CLAD) [29].

In this study, we assess the CRM gene set for use on urine samples from KTx patients, paired with allograft biopsies with known histology, for the non-invasive diagnosis of AR and other immune mediated injuries. Further, we develop a urine CRM (uCRM) score that accurately discriminates between STA and AR patients. We evaluate the clinical potential of this score in detection of bAR by correlating this score with histology scores of tubulitis and interstitial inflammation.

## Materials and methods

### Urine samples and the study cohort

**Fig 1:**
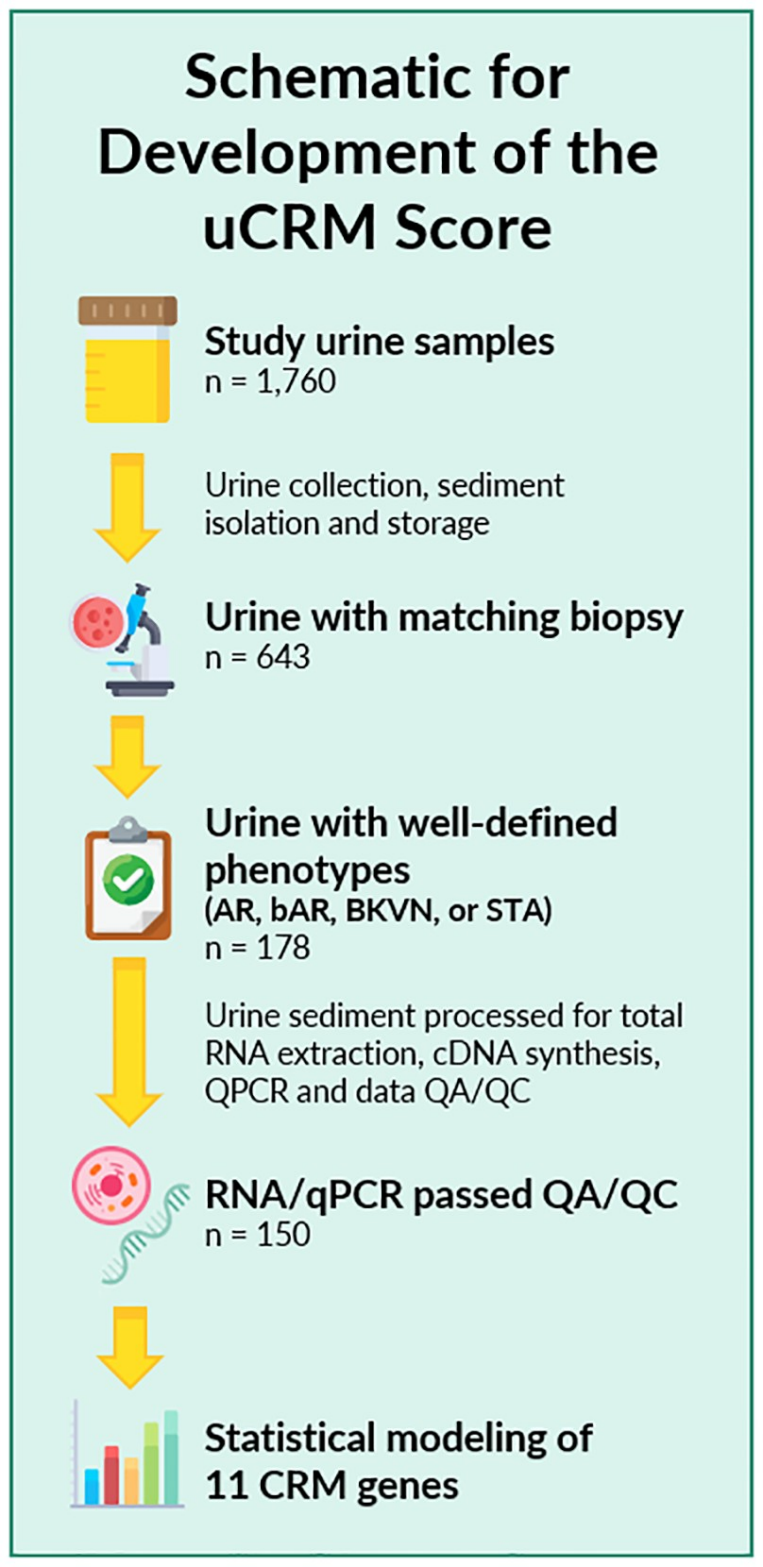
Sample selection and study schematic of the study. 1,760 urine samples were collected between 2000 and 2016, of which 643 had matching biopsy data. 178 of these 643 had well-defined phenotypes of AR, bAR, BKVN, or STA. After RNA extraction, cDNA synthesis, and qPCR quantification, 28 samples did not pass QA/QC, leaving 150 samples for statistical analysis and modeling.

Biobanked urine samples (n = 1760) from KTx recipients enrolled at Stanford University in between 2000 and 2011 and UCSF Medical Center enrolled in between 2014 and 2016 were included in the study. The study was approved by the Institutional Review Board and Ethics Committee of the University of California San Francisco, CA. All patients provided written informed consent to participate in the research, in full adherence to the Declaration of Helsinki. The clinical and research activities being reported are consistent with the Principles of the Declaration of Istanbul as outlined in the Declaration of Istanbul on Organ Trafficking and Transplant Tourism. For urine samples utilized in establishing the uCRM threshold for AR, 178 urine samples were identified with paired kidney allograft biopsies with clearly defined pathologies of either Banff graded AR [30, 31] or no injury/ stable (STA) grafts (Fig 1). In addition, we also evaluated the signature for the uCRM assay in BK viral nephropathy, which is an important confounder for diagnosis of AR and often presents with significant inflammation on the allograft biopsy. Overall, 28 samples were discarded because of QC issues related to low content and poor-quality RNA, resulting in a final count of 150 urine samples from 150 individuals for cross-sectional analysis of immune mediated KTx injury. Each urine sample was matched with a biopsy at the time of urine collection which was evaluated by a central staff pathologist at Stanford University (Richard Sibley) or at UCSF (Zoltan Laszik).

### Patient characteristics

150 unique urine samples were assessed for the uCRM assay in 150 unique kidney transplant patients. Baseline clinical and demographic variables by AR, bAR, BKVN, or STA phenotype are shown in Table 1. There were no significant differences between the groups in the demographic variables, except in recipient age (p = 0.025) and in donor-source (p = 0.0008). These samples were used in cross-sectional analyses for modeling of gene expression data and subsequent development and validation of the uCRM threshold for biopsy-proven AR. Samples were collected from both pediatric (n = 94) and adult (n = 56) patients to enable a model independent of recipient age or baseline immunosuppression. Based on the matched biopsy diagnosis, urine samples were categorized in the following categories: AR (n = 64; 45 biopsies met criteria for Banff confirmed AR with >i2, t2 and infiltration by > 4 mononuclear cells / tubular cross-section, whereas 19 met criteria for borderline AR with i1/ i2 and t0/t1 and infiltration by 1–4 mononuclear cells / tubular cross-section), STA (n = 43), BK virus nephritis (n = 43). Patients received a calcineurin-inhibitor iIS regimen based on tacrolimus and mycophenolate mofetil, with or without steroids, and induction therapy either with Thymoglobulin or anti-IL-2 receptor monoclonal antibody (daclizumab or basiliximab) [32]. Urine samples were obtained at a mean of 731 days post-transplant (range 169–1335 days).

**Table 1:** Demographic characteristics of the study subjects.

	Phenotype				
Characteristic	Stable (N = 43)	Acute Rejection (N = 45)	Borderline Acute Rejection (bAR) (N = 19)	BK Viral Nephropathy (N = 43)	P-value
**Recipient Age—yr**					0.025
Mean ± StdDev	21.9 ± 15.7	22.0 ± 16.5	19.2±13.3	34.3±24.4	
Range	3–58	1–64	3–62	1–79	
**Donor Age—yr**					0.341
Mean ± StdDev	30.0 ± 9.3	33.0 ± 12.0	25.1±13.2	34.8 ± 9.20	
Range	15–47	4–49	4–47	9–61	
Recipient sex—male no. (%)	23 (53)	29 (64)	13 (68)	34 (76)	0.095
Donor sex—male no. (%)	30 (69)	30 (55)	11 (60)	29 (68)	0.836
**Race or ethnic groups—no. (%)**					0.335
White	10 (23)	10 (22)	8 (42)	16 (36)	
Hispanic	23 (54)	21 (47)	9 (47)	13 (30)	
Black	3 (7)	4 (9)	2 (10)	5 (12)	
Asian or Pacific Islander	1 (2)	2 (4)	0 (0)	4 (9)	
Other	6 (14)	8 (18)	0 (0)	5 (12)	
**Indication for renal transplantation—no. (%)**					0.415
Glomerulonephritis	6 (14)	12 (27)	3 (16)	2 (5)	
FSGS	0 (0.0)	0 (0)	1 (5)	1 (2)	
Obstructive Uropathy	4 (9)	2 (4)	0 (0)	3 (7)	
Cystinosis	3 (7)	4 (9)	3 (16)	9 (21)	
Polycystic Kidney Disease	1 (2)	2 (4)	0 (0)	2 (5)	
Reflux Nephropathy	4 (9)	4 (9)	3 (16)	4 (9)	
Dysplasia	2 (5)	1 (2)	1 (5)	0 (0.0)	
Other	23 (53)	20 (44)	8 (42)	22 (51)	
**Donor Source—no. (%)**					0.0008
Living Related	16 (37)	16 (35)	8 (40)	11 (25)	
Living Unrelated	21 (48)	22 (48)	10 (53)	11 (25)	
Deceased Unrelated	6 (15)	7(17)	1 (7)	21(50)	

### Definition of injury phenotypes

All kidney biopsies were blindly and centrally analyzed at each institution by staff pathologists (RS and ZL) and were graded by the Banff classification [31, 33] for acute rejection. Intragraft C4d stains were performed [34] to assess for acute humoral rejection (AHR) [35]. Transplant injury was defined as >20% increase in serum creatinine from its previous steady-state baseline value and an associated biopsy that was either classified as AR or BKVN. AR was defined at minimum, as per Banff schema, a tubulitis score ≥1 accompanied with an interstitial inflammation score ≥1 with both C4d and DSA negative. Both T cell mediated AR (TCMR) and antibody mediated rejection (ABMR) cases were included, though all observed ABMR cases had a mixed phenotype of TCMR and ABMR, as the observance of pure ABMR is rarely observed in low risk, unsensitized cohorts. Borderline changes (bAR) were observed in some cases of TCMR, characterized by infiltration of mononuclear cells (<25% of the parenchyma) or foci of mild tubulitis (1–4 mononuclear cells/tubular cross-section), and for purposes of molecular correlation analysis, these have been shown as bAR, as the burden of histological inflammation was overall lower for these biopsy samples. BKVN was defined as positivity of polyomavirus PCR in peripheral blood (<1000–28,800,000), together with a positive SV40 stain in the concomitant renal allograft biopsy. Normal (STA) allografts were defined by an absence of significant injury pathology on the 6-month protocol biopsy, as defined by Banff schema, stable graft function, no proteinuria and no DSA.

### Urine collection, processing, total RNA extraction, cDNA synthesis, and qPCR

Urine (50 mL; sterile container) was collected from kidney transplant patients before biopsy procedure and prior to any treatment intensification for AR. RNA was extracted from urinary cell sediment following our previously reported protocol [36]. In brief, urine cells were obtained by centrifuging the 50-mL urine specimen at 2000 x g for 20 minutes. RNA was extracted from the urine cell pellets using the RNeasy Plus Micro Kit (Qiagen, Valencia, CA). RNA quality was assessed with the NanoDrop ND-2000 spectrophotometer (ThermoFisher Scientific, Waltham, MA) with 260/280 ratio. cDNA synthesis was performed using 50 ng of extracted RNA using SuperScript VILO^™^ Master Mix (Invitrogen, Carlsbad, CA). qPCR was performed on cDNA synthesized from 50 ng of total RNA, then 1.56 ng of cDNA was processed through specific target amplification and sample dilution with the pooled Taqman assays for the 11 uCRM genes in multiplex, with Taqman PreAmp Master mix (ABI) to 5 μl final volume, for 18 cycles in a thermal cycler, then diluted with DNA Suspension Buffer (TEKnova, CA). Microfluidic qPCR was performed on the 96.96 dynamic array (Fluidigm, South San Francisco, CA) using 2.25 μl of the diluted sample from specific target amplification, along with Taqman Assays (ABI) for each gene transcript **(**S1 Table), Taqman Universal master mix (Applied Biosystems, Foster City, CA) and Loading Reagent (Fluidigm), by priming and loading the chip via the HX IFC Controller and performing qPCR in the BioMark (Fluidgm) system. The relative amount of RNA expression was calculated using a comparative cycle threshold (CT) method. Expression values were normalized to 18S using ribosomal RNA endogenous reference and universal RNA (Agilent Inc., Santa Clara, CA).

### Statistics

All qPCR assays were run in duplicates. All the data are presented as mean ± SEM. For comparisons of the CRM genes per phenotype, a mixed-effects model with the Geisser-Greenhouse correction was used, with multiple comparisons corrections performed using the two-stage linear-step procedure of Benjamini, Krieger, and Yekutieli. Pearson correlation and hierarchical clustering were performed in Morpheus (Broad Institute). For machine learning prediction models, the data was split into a training set (80%) and testing set (20%). A decision tree classification model, validated on the testing set, was used to determine the most accurate uCRM score cut-off. Variable Selection Using Random Forests (VSURF) was used to classify AR vs STA as well as to evaluate and rank individual gene importance. The Random Forest variable importance output is defined as the mean percentage decrease in accuracy of the model if the variable (gene) were excluded (randomly permuted) from the model. Unsupervised clustering to visualize phenotype separation was done using the t-distributed stochastic neighbor embedding algorithm (t-SNE) in Mathematica 11.3 (Wolfram Research, Champaign, IL). Network analysis of the CRM genes was performed using GeneMANIA [37]. Statistics on demographic variables were performed using Chi-square analyses for discrete and the Kruskal-Wallis test for continuous variables in JMP 14.2 (SAS Institute, Cary, NC). Unless otherwise stated, all other analyses were performed and visualized with Prism 8.0.1 (GraphPad, Carlsbad, CA).

### Study approval

The study was approved by the ethics committees of both Stanford University Medical School and UCSF Medical Center. All adult patients and parents/guardians of non-adult patients provided written informed consent to participate in the research, in full adherence to the Declaration of Helsinki. The clinical and research activities being reported are consistent with the Principles of the Declaration of Istanbul as outlined in the Declaration of Istanbul on Organ Trafficking and Transplant Tourism.

## Results

Baseline clinical and demographic variables for all 150 KTx recipients with AR, bAR, BKVN, and STA phenotypes are shown in Table 1.

### Relative abundance, and correlation of CRM gene expression in the urine cell sediment

#### Relative abundance

To determine relative abundance of the CRM gene transcripts in the urine sediments, the cycle threshold (Ct) values were used as a metric of abundance. The lower the Ct value, the higher its abundance among the CRM genes. Among the 11 CRM genes, BASP1 was the most abundant transcript in the urinary cell sediment. BASP1 was followed by TAP1, PSMB9, and ISG20 as the 4 topmost abundant transcripts. LCK and CD6 were among the least abundant transcripts in the urine sediments in the CRM gene-set. Since the Ct values ranged from the lowest Ct value of 14 to the highest Ct value of 20, there was a 64-fold difference between the BASP1 and CD6 gene transcripts, with CD6 being the least abundant transcript.

#### Correlation of gene expression among CRM gene genes

**Fig 2:**
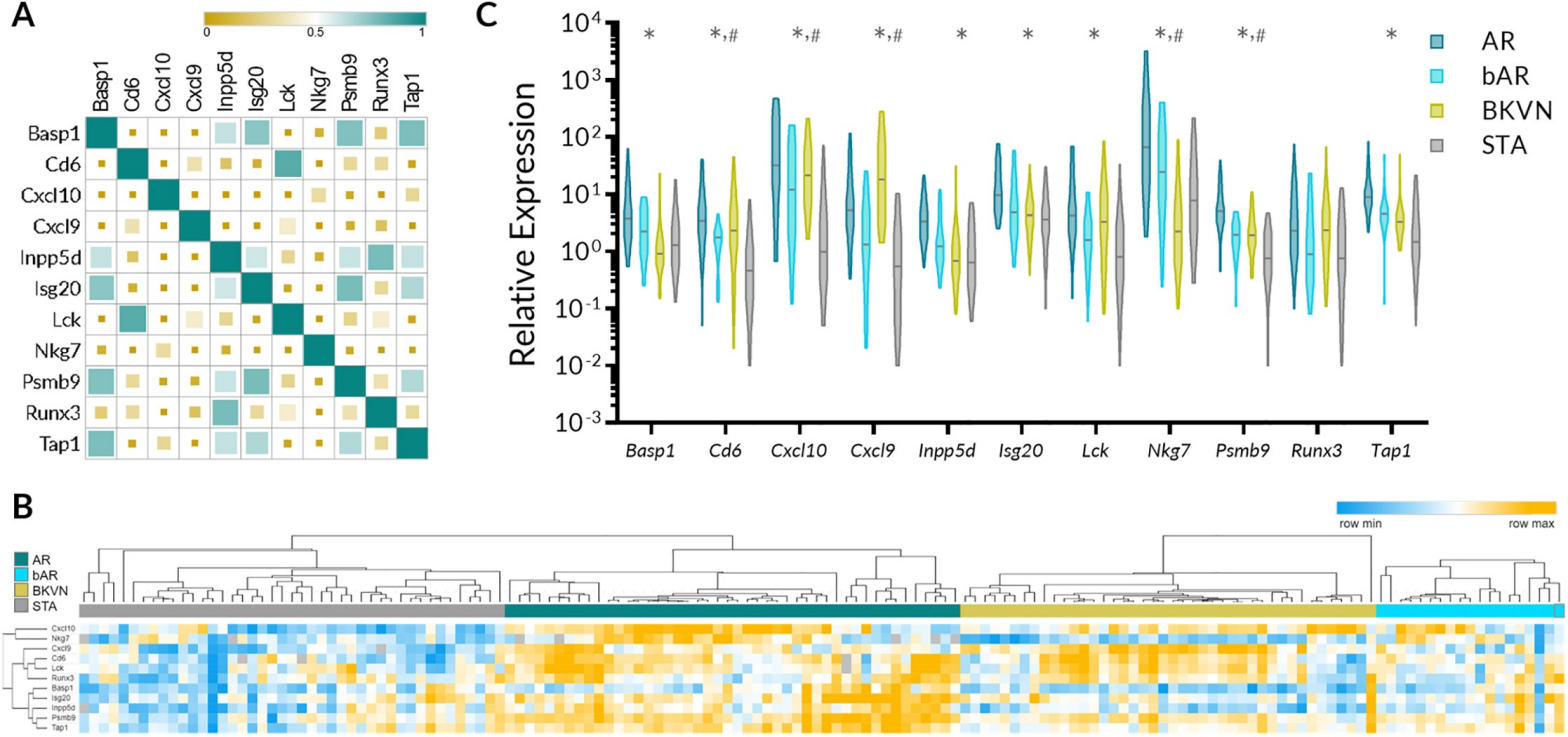
Relative abundance and correlation of abundance of CRM genes in the urine and expression of CRM genes across different clinical phenotypes of kidney transplantation. A. Pearson correlation matrix demonstrating correlation among 11 CRM genes in their expression in urinary cell sediments. The size of the square serves as a visual indicator of the strength of the correlation. B. Heatmap with supervised clustering by phenotype demonstrating relative expression of CRM genes in AR, bAR, BKVN, and STA. C. Violin plots depicting the distribution of the CRM genes in AR, bAR, BKVN, and STA urine cell pellet. * indicates that AR vs STA was significant after multiple comparisons. ^#^ indicates that bAR vs STA was significant after multiple comparisons. Additional statistics are available in Table 2.

Next, we evaluated correlation of gene expression among 11 CRM genes. The correlation ranged from very weak (r = -0.17 for CXCL9 and NKG7 and r = -0.10 CXCL10 and RUNX3) to very strong (r = 0.77 for INPP5D and TAP1 and the same value for CD6 and LCK). Although CXCL9 and CXCL10 are in the same class of chemokines, the gene expression correlation between them was only moderate (r = 0.46). The graphical presentation of correlation matrix is presented in Fig 2A. A heatmap generated using supervised clustering demonstrates considerable increase in gene expression values of the CRM genes in AR, bAR, and BKVN compared to STA phenotype **(**Fig 2B).

### uCRM gene expression in urinary sediments with biopsy confirmed AR and BKVN

#### Gene expression of CRM genes in AR and bAR

Next, we evaluated gene expression of each of 11 CRM genes for their relative expression in AR, bAR, BKVN, and STA. A summary of results of the analysis is presented in Table 2 and Fig 2C. 10 out of the 11 genes were significantly increased in AR urine sediments when compared to urine sediments from STA. However, only five CRM genes (Cd6, Cxcl10, Cxcl9, Nkg7, and Psmb9) were significantly upregulated in bAR samples compared to STA and their expression in the bAR group was relatively lower than in Banff graded AR group, highlighting that the uCRM genes can reflect the inflammatory burden within the allograft Fig 2C.

**Table 2:** Gene expression levels of CRM genes across different phenotypes.

	Basp1	Cd6	Cxcl10	Cxcl9	Inpp5d	Isg20	Lck	Nkg7	Psmb9	Runx3	Tap1
**AR mean**	7.61	5.78	109.97	11.01	4.87	18.16	10.74	352.94	7.67	6.37	13.95
**AR STDEV**	11.01	7.22	137.88	15.45	4.65	17.74	15.41	712.72	7.52	11.95	14.99
**STA mean**	2.52	0.84	5.05	1.54	1.48	5.19	2.24	25.95	1.13	2.03	3.31
**STA STDEV**	3.46	1.40	11.84	2.46	1.73	5.81	5.20	43.89	1.02	2.76	4.38
**AR vs STA q value**	**0.0120**	**<0.0001**	**<0.0001**	**<0.0001**	**0.0004**	**0.0004**	**<0.0001**	**0.0160**	**<0.0001**	0.1126	**<0.0001**
**bAR mean**	3.08	1.55	39.52	4.52	2.26	10.27	2.45	82.63	2.06	3.42	7.15
**bAR STDEV**	2.61	1.05	55.42	7.54	2.93	14.69	2.75	121.31	1.26	5.78	10.69
**bAR vs STA q value**	0.1872	**0.0477**	**0.0011**	**0.0167**	0.2361	0.1087	0.6258	**0.0413**	**0.0034**	0.4405	0.1034
**BKVN mean**	1.76	5.75	39.29	52.07	1.73	5.98	9.23	7.69	2.69	5.14	5.15
**BKVN STDEV**	3.59	9.52	48.36	73.99	4.76	6.69	16.82	16.39	2.35	10.59	7.66
**AR vs BKVN q value**	**0.0067**	0.1198	**0.0005**	**0.0008**	**0.0063**	**0.0004**	0.1977	**0.0117**	**<0.0001**	0.5289	**0.0002**
**BKVN vs STA q value**	0.1795	**0.0021**	**<0.0001**	**<0.0001**	0.4197	0.3301	**0.0352**	**0.0270**	**0.0001**	0.2205	0.1862

#### Gene expression of CRM genes in BKVN

Expression of CD6, CXCL10, CXCL9, LCK, NKG7, and PSMB9 were differentially regulated in urine samples with patients with BKVN when compared to the samples from STA patients. Out of the six genes with statistically different gene expression values between BKVN and STA samples, expression of only NKG7 was substantially lower in BKVN urine.

### Determination of a urine CRM (uCRM) gene expression score to identify kidney transplant rejection

**Fig 3:**
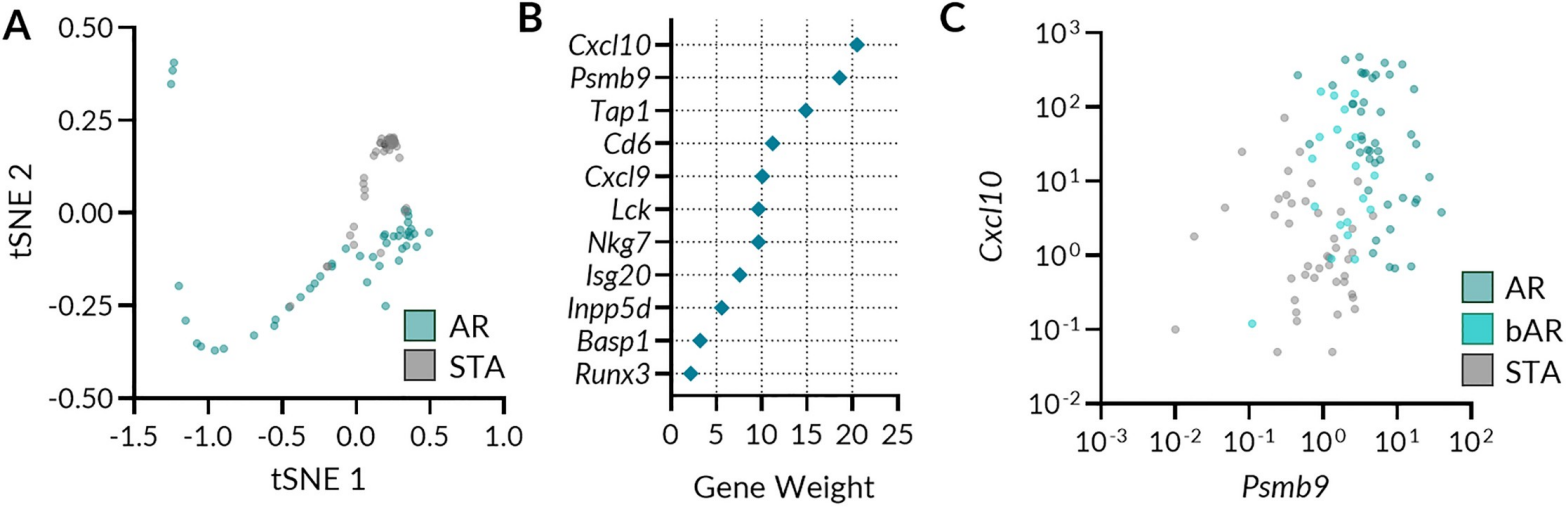
Performance evaluation of uCRM genes in phenotype discrimination. A. Unsupervised clustering of AR and STA gene expression data using T-distributed Stochastic Neighbor Embedding (t-SNE) with a perplexity value of 78. B. Use of Variable Selection Using Random Forests (VSURF) to identify genes that have high importance in AR detection. C. Distribution of transplant outcomes in terms of AR, bAR, and STA on the two VSURF-selected genes Cxcl10 and Psmb9.

Because expression of the CRM gene-set was not homogenous across the transplant phenotypes and there was substantial physiological cross-talk between the different genes (S1 Fig), we used nonlinear supervised methods to further differentiate and classify phenotypes. Unsupervised clustering via t-SNE was performed to determine relationships between the uCRM genes and phenotypes. Fig 3A shows the t-SNE plot, indicating that the 11 CRM genes could almost entirely segregate AR from STA samples. The VSURF model, dependent on Random Forests importance scores, determined that PSMB9 and CXCL10 were the two most important genes in classifying AR from STA. Fig 3B further depicts the significance of these 2 genes. The importance plot of gene weights denotes that either of the 2 genes, if excluded from the model, corresponds to an approximate 20% decrease in accuracy of the model. These two genes could classify AR versus STA with almost as high an accuracy as the 11 gene model, with a sensitivity of 93.6% and specificity of 97.6%. Gene expression thresholds for these two genes were determined by a decision tree classifier and the log-scale values of these two genes are depicted in Fig 3C. A threshold of 28 for CXCL10 and 3 for PSMB9 correctly classifies 86/88 AR & STA cases for an overall accuracy of 97.7%. Notably, bAR samples fell between the AR and STA phenotypes, suggesting the gradation of these two genes in the degree of allograft inflammation.

**Fig 4:**
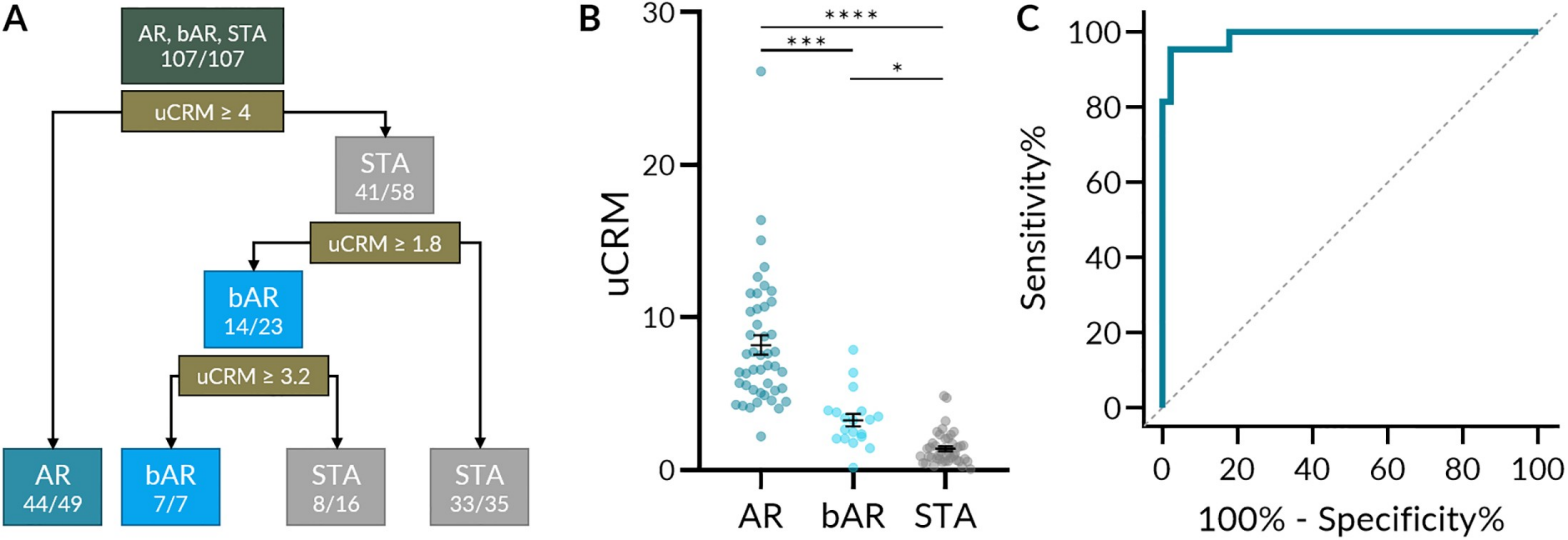
uCRM score classification performance and threshold development by decision tree. A. A decision tree based on uCRM score classification performance and threshold that could correctly classify AR, bAR, and STA with 96.6% accuracy (85/88). B. Scatter dot plot of the uCRM score for AR, bAR, and STA phenotypes. Significance was determined by nonparametric Kruskal-Wallis test with Dunn’s multiple comparisons correction. C. ROC curve of the uCRM score in discriminating between AR and STA phenotypes (AUC = 0.9886, P < 0.0001). * < 0.05. *** P < 0.001. **** P < 0.0001.

To explore the classification performance of the uCRM score on AR, borderline AR, and STA cases, a decision tree classifier was produced (Fig 4A). The decision tree determined optimal uCRM score thresholds for each phenotype. A score greater than 4 correctly classified 44/49 AR cases; a score less than 1.8 correctly classified 33/35 STA cases. 14/23 borderline cases were between these two thresholds. The distribution of uCRM scores by phenotype is depicted in Fig 4B. The mean uCRM scores (SEM) for AR, bAR, and STA were 8.195 (0.631), 3.265 (0.4120), and 1.404 (0.162) respectively, and all comparisons were significant after multiple comparisons correction. The uCRM score could distinguish between AR and STA with high accuracy—at a threshold of 3.63, the sensitivity and specificity were 95.35% and 97.78% respectively (Fig 4C). When distinguishing between AR and the combination of bAR and STA, the uCRM score retained a high accuracy—at the same threshold, the sensitivity and specificity were 87.10% and 97.78% respectively (S2A Fig).

When including the BKVN samples, all phenotypes were significantly different from one another after multiple comparisons correction except bAR and BKVN (S2B Fig). When distinguishing between AR and the combination of bAR, STA, and BKVN, the uCRM score still retained a high, but lower accuracy. Using the same 3.63 threshold, the sensitivity and specificity were 76.92% and 97.78% respectively (S2C Fig).

### The uCRM score correlates with AR specific biopsy histological lesions

**Fig 5:**
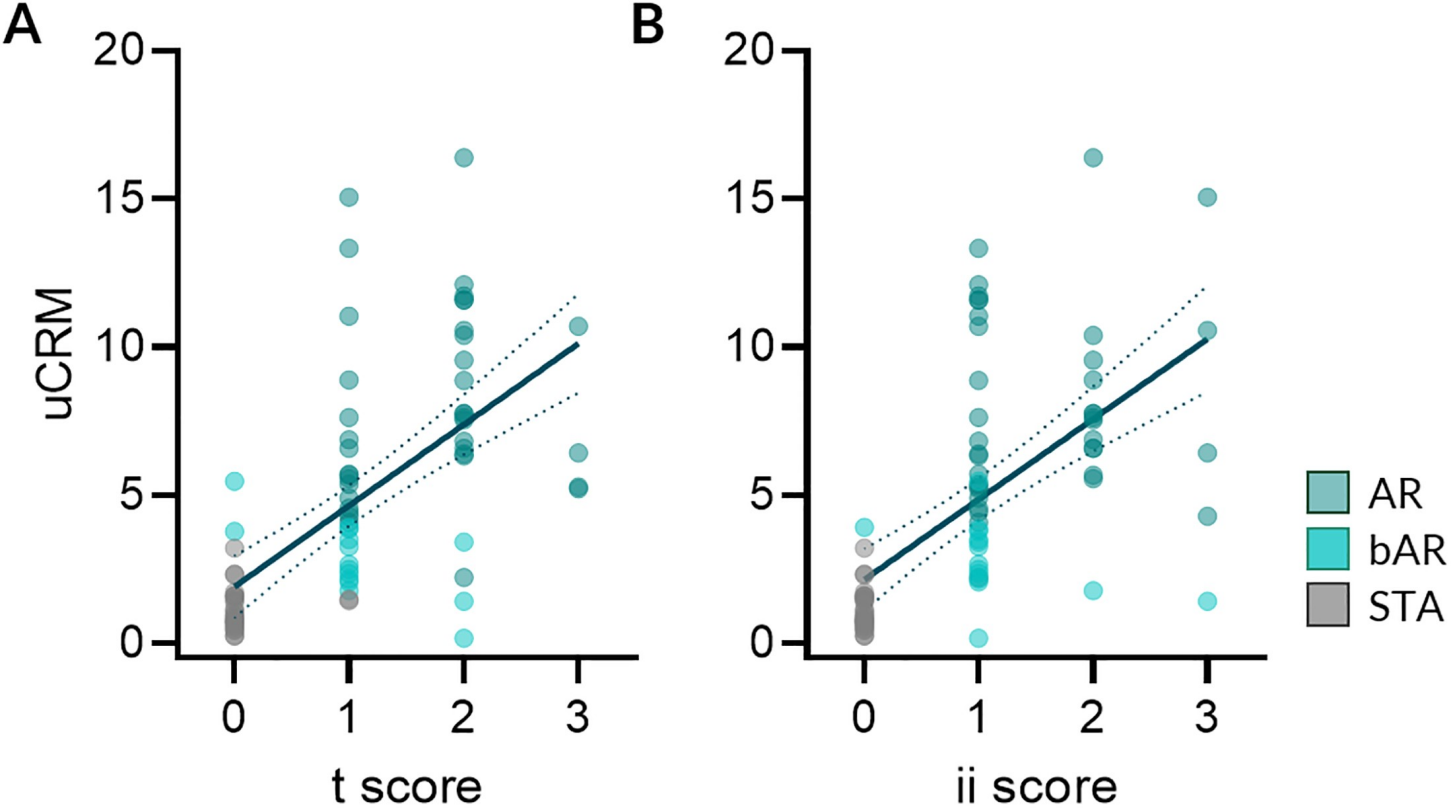
The uCRM score correlates with the extent of AR lesions. The uCRM scores were correlated with the extent of the acute allograft lesions. A. Correlation between the tubular (t) scores and the uCRM scores (R = 0.5479, P < 0.0001) and B. Correlation between the interstitial inflammation (ii) scores and the uCRM scores (R = 0.4420, P < 0.0001). Dotted lines depict the 95% confidence interval of the regression line.

Notably, the trend of increasing uCRM score from STA to bAR to AR suggested that the uCRM score could detect gradations of inflammation that were clinically relevant. As such, we evaluated whether the uCRM score was associated with the extent of histological AR lesions observed in matched biopsies from the same patient, collected simultaneously. As seen in Fig 5A and 5B, the uCRM scores correlated with the extent of the tubulitis (t) and the interstitial inflammation (ii) biopsy scores in AR (R = 0.5479, P < 0.0001 and R = 0.4420, P < 0.0001 for the uCRM score regarding t and ii, respectively). There was no correlation between the uCRM score and measures of tubular atrophy (ta), glomerulosclerosis (gs), mesangial matrix (mm), intimal proliferation (cv), medial arteriolar hyaline (ah), tubular vacuolization (tv), arteritis (v), or acute glomerulitis (g) (S3 Fig).

## Discussion

There is an urgent need in transplant medicine for developing reliable and non-invasive monitoring tools that may help transplant clinicians predict the risk of allograft injury, preferentially before allograft damage has already been established. While a number of transcriptional biomarkers have been associated to AR, most of the studies have basically focused on a unique or single transcriptional factor and do not reflect the entire molecular complexity of the biological process of allograft rejection [11, 38]. Moreover, while the current gold standard for diagnosing the presence of immune-mediated allograft injury is the allograft biopsy, it is well known that the procedure possesses key limitations in terms of the frequent erratic sampling representation, its high cost, and the impracticality for repetitive screening due to the invasive nature of the technique.

Several reports have shown the value of studying different biomarkers predicting AR in urine samples of kidney transplant recipients [20–22]. Increased urinary levels of immune effector molecules and transcripts such as granzyme B, CXCL10, CXCL9, IFN-γ, and CXCR3, have been shown to be highly associated to AR and in some cases, even predict the advent of AR in advance [19, 24, 39–43]. Taking advantage of recently reported data by our group [27, 29, 44] showing a common rejection module of gene expression in allograft biopsies during AR, irrespective of the type of tissue organ, the main goal of this study was to investigate whether the assessment of the CRM in the urine of kidney transplant patients could be useful as an ideal non-invasive biomarker predicting the advent of AR.

While many of the individual CRM genes and gene products have been assessed individually, this is the first report of the collective, noninvasive use of the CRM genes in the prediction of AR in KTx. For example, urinary CXCL9 mRNA and protein and CXCL10 mRNA had previously been evaluated in multicenter studies for the diagnosis of AR [13, 45, 46]. PSMB9 transcripts in renal biopsies had also been previously associated with graft quality and the prediction of acute rejection [47].

We have analyzed gene expression data on urine sediments from KTx patients for the relative abundance of CRM transcripts and their correlation of expression among CRM genes **(**Fig 2A). In line with our previously reported study analyzing the CRM score in tissue kidney and lung allograft samples, the CRM genes had increased expression in AR and other transplant injuries such as bAR and BKVN **(**Fig 2B and 2C). In this report, we also observed a strong correlation between the newly developed uCRM score and histological inflammatory scores (t and ii scores of kidney biopsies). Since most of these CRM genes are expressed almost exclusively in infiltrating immune cells, the increased expression of CRM genes in urine sediments suggests that there is an increased release of infiltrating immune cells in the urine of kidney transplant recipients undergoing graft injury.

Next, we utilized a combined score calculated from individual gene expression values of individual CRM genes, the uCRM score, as a metric to classify kidney transplant patients into either patient with acute rejection or no-injury and determined a threshold for AR. The results from this study demonstrate the power of uCRM assay in not only identifying patients with AR, but also quantifying the degree of injury taking place in the allograft, as the score increases from low values in STA patients, to intermediate values in bAR patients, and to high values in AR patients, as reflected in the tubulitis and interstitial inflammation histology scores. We believe that the uCRM score has potential utility in transplant monitoring and may serve as an adjunct to or referring test for biopsies. A patient with a low uCRM score may be able to avoid unnecessary protocol biopsies while a patient with a high uCRM score may require serial monitoring or a for-cause biopsy to assess graft status.

We acknowledge several limitations of this study that includes (i) limited sample size of the study, (ii) absence of other transplant injury phenotypes such as chronic allograft injury or drug toxicity, and (iii) lack of assessment of uCRM score in a longitudinal sample in a larger cohort size. These promising findings suggest that additional, prospective studies are needed to validate and fully assess the potential utility of the uCRM score in the clinical setting. In summary, we present a non-invasive, urine-based biomarker developed from a Common Rejection Module composed of 11 genes that can identify transplant injury and rejection in kidney transplant patients.

## Supporting information

S1 Fig Interaction network analysis of CRM genes. A network view of the CRM genes. Cross-hatched bubbles are the CRM genes while solid bubbles indicate partners identified through network analysis. Connections are color-coded based on the type of interaction. Red, physical interaction; purple, co-expression; orange, predicted; blue, co-localization; turquoise, pathway; green, genetic interactions; yellow, shared protein domains.
(PDF)

S2 Fig uCRM score classification performance inclusive of BKVN phenotype. A. ROC curve of the uCRM score in discriminating between AR and the combination of bAR and STA phenotypes (AUC = 0.9677, P < 0.0001). B. Scatter dot plot of the uCRM score for AR, bAR, STA, and BKVN phenotypes. Significance was determined by nonparametric Kruskal-Wallis test with Dunn’s multiple comparisons correction. C. ROC curve of the uCRM score in discriminating between AR and the combination of bAR and STA phenotypes (AUC = 0.9111, P < 0.0001). * < 0.05. ** < 0.01. *** P < 0.001. **** P < 0.0001.
(PDF)

S3 Fig Lack of association of uCRM score with additional histological parameters. The correlation between the uCRM score and various histological parameters was determined. Other than the tubulitis (t) and interstitial inflammation (ii) scores that were significantly correlated (Fig 5A and 5B), none of tubular atrophy (ta), glomerulosclerosis (gs), mesangial matrix (mm), intimal proliferation (cv), medial arteriolar hyaline (ah), tubular vacuolization (tv), arteritis (v), ora cute glomerulitis (g) scores were significantly correlated.
(PDF)

S1 Table Primer information for primers used for uCRM assay. (PDF)

S1 Dataset Deidentified RQ values for the CRM genes and the uCRM Score. (XLSX)

## References

[1] Wolfe RA, Ashby VB, Milford EL, Ojo AO, Ettenger RE, Agodoa LY, et al Comparison of mortality in all patients on dialysis, patients on dialysis awaiting transplantation, and recipients of a first cadaveric transplant. The New England journal of medicine. 1999;341(23):1725–30. 10.1056/NEJM199912023412303 . 10580071

[2] Laupacis A, Keown P, Pus N, Krueger H, Ferguson B, Wong C, et al A study of the quality of life and cost-utility of renal transplantation. Kidney international. 1996;50(1):235–42. . 8807593 10.1038/ki.1996.307

[3] Lodhi SA, Lamb KE, Meier-Kriesche HU. Improving long-term outcomes for transplant patients: making the case for long-term disease-specific and multidisciplinary research. American journal of transplantation: official journal of the American Society of Transplantation and the American Society of Transplant Surgeons. 2011;11(10):2264–5. 10.1111/j.1600-6143.2011.03713.x . 21957938

[4] Naesens M, Khatri P, Li L, Sigdel TK, Vitalone MJ, Chen R, et al Progressive histological damage in renal allografts is associated with expression of innate and adaptive immunity genes. Kidney international. 2011;80(12):1364–76. 10.1038/ki.2011.245 . 21881554 PMC4492284

[5] Sigdel TK, Li L, Tran TQ, Khatri P, Naesens M, Sansanwal P, et al Non-HLA antibodies to immunogenic epitopes predict the evolution of chronic renal allograft injury. Journal of the American Society of Nephrology: JASN. 2012;23(4):750–63. 10.1681/ASN.2011060596 . 22302197

[6] Pape L, Offner G, Ehrich JH, de Boer J, Persijn GG. Renal allograft function in matched pediatric and adult recipient pairs of the same donor. Transplantation. 2004;77(8):1191–4. . 15114083 10.1097/01.tp.0000120099.92220.7a

[7] Provoost AP, Wolff ED, de Keijzer MH, Molenaar JC. Influence of the recipient’s size upon renal function following kidney transplantation. An experimental and clinical investigation. Journal of pediatric surgery. 1984;19(1):63–7. 10.1016/s0022-3468(84)80018-4 . 6366181

[8] Moreso F, Lopez M, Vallejos A, Giordani C, Riera L, Fulladosa X, et al Serial protocol biopsies to quantify the progression of chronic transplant nephropathy in stable renal allografts. American journal of transplantation: official journal of the American Society of Transplantation and the American Society of Transplant Surgeons. 2001;1(1):82–8. . 12095044 10.1034/j.1600-6143.2001.010115.x

[9] Davis ID, Oehlenschlager W, O’Riordan MA, Avner ED. Pediatric renal biopsy: should this procedure be performed in an outpatient setting? Pediatric nephrology. 1998;12(2):96–100. . 9543363 10.1007/s004670050412

[10] Roedder S, Sigdel T, Salomonis N, Hsieh S, Dai H, Bestard O, et al The kSORT assay to detect renal transplant patients at high risk for acute rejection: results of the multicenter AART study. PLoS medicine. 2014;11(11):e1001759 10.1371/journal.pmed.1001759 . 25386950 PMC4227654

[11] Sarwal M, Chua MS, Kambham N, Hsieh SC, Satterwhite T, Masek M, et al Molecular heterogeneity in acute renal allograft rejection identified by DNA microarray profiling. The New England journal of medicine. 2003;349(2):125–38. 10.1056/NEJMoa035588 . 12853585

[12] Sigdel TK, Sarwal MM. Recent advances in biomarker discovery in solid organ transplant by proteomics. Expert review of proteomics. 2011;8(6):705–15. 10.1586/epr.11.66 . 22087656 PMC3282122

[13] Suthanthiran M, Schwartz JE, Ding R, Abecassis M, Dadhania D, Samstein B, et al Urinary-cell mRNA profile and acute cellular rejection in kidney allografts. The New England journal of medicine. 2013;369(1):20–31. 10.1056/NEJMoa1215555 . 23822777 PMC3786188

[14] Sigdel TK, Gao Y, He J, Wang A, Nicora CD, Fillmore TL, et al Mining the human urine proteome for monitoring renal transplant injury. Kidney international. 2016;89(6):1244–52. 10.1016/j.kint.2015.12.049 . 27165815 PMC5221536

[15] Sigdel TK, Ng YW, Lee S, Nicora CD, Qian WJ, Smith RD, et al Perturbations in the urinary exosome in transplant rejection. Frontiers in medicine. 2014;1:57 10.3389/fmed.2014.00057 . 25593928 PMC4292055

[16] Yang JY, Sarwal MM. Transplant genetics and genomics. Nature reviews Genetics. 2017;18(5):309–26. 10.1038/nrg.2017.12 . 28286337

[17] Loupy A, Lefaucheur C, Vernerey D, Chang J, Hidalgo LG, Beuscart T, et al Molecular microscope strategy to improve risk stratification in early antibody-mediated kidney allograft rejection. Journal of the American Society of Nephrology: JASN. 2014;25(10):2267–77. Epub 2014/04/05. 10.1681/ASN.2013111149 . 24700874 PMC4178445

[18] Sigdel TK, Vitalone MJ, Tran TQ, Dai H, Hsieh SC, Salvatierra O, et al A rapid noninvasive assay for the detection of renal transplant injury. Transplantation. 2013;96(1):97–101. . 23756769 10.1097/TP.0b013e318295ee5a PMC4472435

[19] Hauser IA, Spiegler S, Kiss E, Gauer S, Sichler O, Scheuermann EH, et al Prediction of acute renal allograft rejection by urinary monokine induced by IFN-gamma (MIG). Journal of the American Society of Nephrology: JASN. 2005;16(6):1849–58. 10.1681/ASN.2004100836 . 15857922

[20] Hu H, Kwun J, Aizenstein BD, Knechtle SJ. Noninvasive detection of acute and chronic injuries in human renal transplant by elevation of multiple cytokines/chemokines in urine. Transplantation. 2009;87(12):1814–20. . 19543058 10.1097/TP.0b013e3181a66b3e

[21] Jackson JA, Kim EJ, Begley B, Cheeseman J, Harden T, Perez SD, et al Urinary chemokines CXCL9 and CXCL10 are noninvasive markers of renal allograft rejection and BK viral infection. American journal of transplantation: official journal of the American Society of Transplantation and the American Society of Transplant Surgeons. 2011;11(10):2228–34. 10.1111/j.1600-6143.2011.03680.x . 21812928 PMC3184377

[22] Schaub S, Nickerson P, Rush D, Mayr M, Hess C, Golian M, et al Urinary CXCL9 and CXCL10 levels correlate with the extent of subclinical tubulitis. American journal of transplantation: official journal of the American Society of Transplantation and the American Society of Transplant Surgeons. 2009;9(6):1347–53. 10.1111/j.1600-6143.2009.02645.x . 19459809

[23] Segerer S, Cui Y, Eitner F, Goodpaster T, Hudkins KL, Mack M, et al Expression of chemokines and chemokine receptors during human renal transplant rejection. American journal of kidney diseases: the official journal of the National Kidney Foundation. 2001;37(3):518–31. . 11228176

[24] Tatapudi RR, Muthukumar T, Dadhania D, Ding R, Li B, Sharma VK, et al Noninvasive detection of renal allograft inflammation by measurements of mRNA for IP-10 and CXCR3 in urine. Kidney international. 2004;65(6):2390–7. 10.1111/j.1523-1755.2004.00663.x . 15149352

[25] Menon MC, Murphy B, Heeger PS. Moving Biomarkers toward Clinical Implementation in Kidney Transplantation. JASN. 2017. 10.1681/ASN.2016080858 PMC5328171 28062570

[26] Naesens M, Anglicheau D. Precision Transplant Medicine: Biomarkers to the Rescue. JASN. 2017. 10.1681/ASN.2017010004 PMC5748900 28993504

[27] Khatri P, Roedder S, Kimura N, De Vusser K, Morgan AA, Gong Y, et al A common rejection module (CRM) for acute rejection across multiple organs identifies novel therapeutics for organ transplantation. The Journal of experimental medicine. 2013;210(11):2205–21. 10.1084/jem.20122709 . 24127489 PMC3804941

[28] Sigdel TK, Bestard O, Tran TQ, Hsieh SC, Roedder S, Damm I, et al A Computational Gene Expression Score for Predicting Immune Injury in Renal Allografts. PloS one. 2015;10(9):e0138133 10.1371/journal.pone.0138133 . 26367000 PMC4569485

[29] Sacreas A, Yang JYC, Vanaudenaerde BM, Sigdel TK, Liberto JM, Damm I, et al The common rejection module in chronic rejection post lung transplantation. PloS one. 2018;13(10):e0205107 Epub 2018/10/06. 10.1371/journal.pone.0205107 . 30289917 PMC6173434

[30] Sis B, Mengel M, Haas M, Colvin RB, Halloran PF, Racusen LC, et al Banff '09 meeting report: antibody mediated graft deterioration and implementation of Banff working groups. American journal of transplantation: official journal of the American Society of Transplantation and the American Society of Transplant Surgeons. 2010;10(3):464–71. 10.1111/j.1600-6143.2009.02987.x . 20121738

[31] Solez K, Colvin RB, Racusen LC, Haas M, Sis B, Mengel M, et al Banff 07 classification of renal allograft pathology: updates and future directions. American journal of transplantation: official journal of the American Society of Transplantation and the American Society of Transplant Surgeons. 2008;8(4):753–60. 10.1111/j.1600-6143.2008.02159.x . 18294345

[32] Sarwal MM, Ettenger RB, Dharnidharka V, Benfield M, Mathias R, Portale A, et al Complete steroid avoidance is effective and safe in children with renal transplants: a multicenter randomized trial with three-year follow-up. American journal of transplantation: official journal of the American Society of Transplantation and the American Society of Transplant Surgeons. 2012;12(10):2719–29. 10.1111/j.1600-6143.2012.04145.x . 22694755 PMC3681527

[33] Racusen LC. The Banff schema and differential diagnosis of allograft dysfunction. Transplantation proceedings. 2004;36(3):753–4. 10.1016/j.transproceed.2004.03.031 . 15110651

[34] Jianghua C, Wenqing X, Huiping W, Juan J, Jianyong W, Qiang H. C4d as a significant predictor for humoral rejection in renal allografts. Clinical transplantation. 2005;19(6):785–91. 10.1111/j.1399-0012.2005.00422.x . 16313326

[35] Crespo M, Pascual M, Tolkoff-Rubin N, Mauiyyedi S, Collins AB, Fitzpatrick D, et al Acute humoral rejection in renal allograft recipients: I. Incidence, serology and clinical characteristics. Transplantation. 2001;71(5):652–8. . 11292296 10.1097/00007890-200103150-00013

[36] Keslar KS, Lin M, Zmijewska AA, Sigdel TK, Tran TQ, Ma L, et al Multicenter Evaluation of a Standardized Protocol for Noninvasive Gene Expression Profiling. AJT. 2013. 10.1111/ajt.12284 PMC3781926 23802725

[37] Montojo J, Zuberi K, Rodriguez H, Bader GD, Morris Q. GeneMANIA: Fast gene network construction and function prediction for Cytoscape. F1000Research. 2014;3:153 10.12688/f1000research.4572.1 . 25254104 PMC4168749

[38] Der SD, Zhou A, Williams BR, Silverman RH. Identification of genes differentially regulated by interferon alpha, beta, or gamma using oligonucleotide arrays. Proceedings of the National Academy of Sciences of the United States of America. 1998;95(26):15623–8. 10.1073/pnas.95.26.15623 . 9861020 PMC28094

[39] Hartono C, Muthukumar T, Suthanthiran M. Noninvasive diagnosis of acute rejection of renal allografts. Current opinion in organ transplantation. 2010;15(1):35–41. 10.1097/MOT.0b013e3283342728 . 19935064 PMC2879051

[40] Lazzeri E, Rotondi M, Mazzinghi B, Lasagni L, Buonamano A, Rosati A, et al High CXCL10 expression in rejected kidneys and predictive role of pretransplant serum CXCL10 for acute rejection and chronic allograft nephropathy. Transplantation. 2005;79(9):1215–20. . 15880073 10.1097/01.tp.0000160759.85080.2e

[41] Matz M, Beyer J, Wunsch D, Mashreghi MF, Seiler M, Pratschke J, et al Early post-transplant urinary IP-10 expression after kidney transplantation is predictive of short- and long-term graft function. Kidney international. 2006;69(9):1683–90. 10.1038/sj.ki.5000343 . 16572110

[42] Simon T, Opelz G, Wiesel M, Ott RC, Susal C. Serial peripheral blood perforin and granzyme B gene expression measurements for prediction of acute rejection in kidney graft recipients. American journal of transplantation: official journal of the American Society of Transplantation and the American Society of Transplant Surgeons. 2003;3(9):1121–7. . 12919092 10.1034/j.1600-6143.2003.00187.x

[43] Yannaraki M, Rebibou JM, Ducloux D, Saas P, Duperrier A, Felix S, et al Urinary cytotoxic molecular markers for a noninvasive diagnosis in acute renal transplant rejection. Transplant international: official journal of the European Society for Organ Transplantation. 2006;19(9):759–68. 10.1111/j.1432-2277.2006.00351.x . 16918537

[44] Yang JYC, Verleden SE, Zarinsefat A, Vanaudenaerde BM, Vos R, Verleden GM, et al Cell-Free DNA and CXCL10 Derived from Bronchoalveolar Lavage Predict Lung Transplant Survival. J Clin Med. 2019;8(2). Epub 2019/02/20. 10.3390/jcm8020241 . 30781765 PMC6406976

[45] Faddoul G, Nadkarni GN, Bridges ND, Goebel J, Hricik DE, Formica R, et al Analysis of Biomarkers Within the Initial 2 Years Posttransplant and 5-Year Kidney Transplant Outcomes: Results From Clinical Trials in Organ Transplantation-17. Transplantation. 2018;102(4):673–80. Epub 2017/12/01. 10.1097/TP.0000000000002026 . 29189482 PMC6018026

[46] Hricik DE, Nickerson P, Formica RN, Poggio ED, Rush D, Newell KA, et al Multicenter validation of urinary CXCL9 as a risk-stratifying biomarker for kidney transplant injury. American journal of transplantation: official journal of the American Society of Transplantation and the American Society of Transplant Surgeons. 2013;13(10):2634–44. Epub 2013/08/24. 10.1111/ajt.12426 . 23968332 PMC3959786

[47] Kotsch K, Kunert K, Merk V, Reutzel-Selke A, Pascher A, Fritzsche F, et al Novel markers in zero-hour kidney biopsies indicate graft quality and clinical outcome. Transplantation. 2010;90(9):958–65. Epub 2010/09/23. . 20859252 10.1097/TP.0b013e3181f546e8

