# Supplementary material for: A urinary Common Rejection Module (uCRM) score for non-invasive kidney transplant monitoring
Source: PLoS One. 2019 Jul 31;14(7):e0220052. doi: 10.1371/journal.pone.0220052 (PMC6668802; doi:10.1371/journal.pone.0220052)

Supplemental Figure S1. Interaction network analysis of CRM genes. A network view of the CRM genes. Cross-hatched bubbles are the CRM genes while solid bubbles indicate partners identified through network analysis. Connections are color-coded based on the type of interaction. Red, physical interaction; purple, co-expression; orange, predicted; blue, co-localization; turquoise, pathway; green, genetic interactions; yellow, shared protein domains.

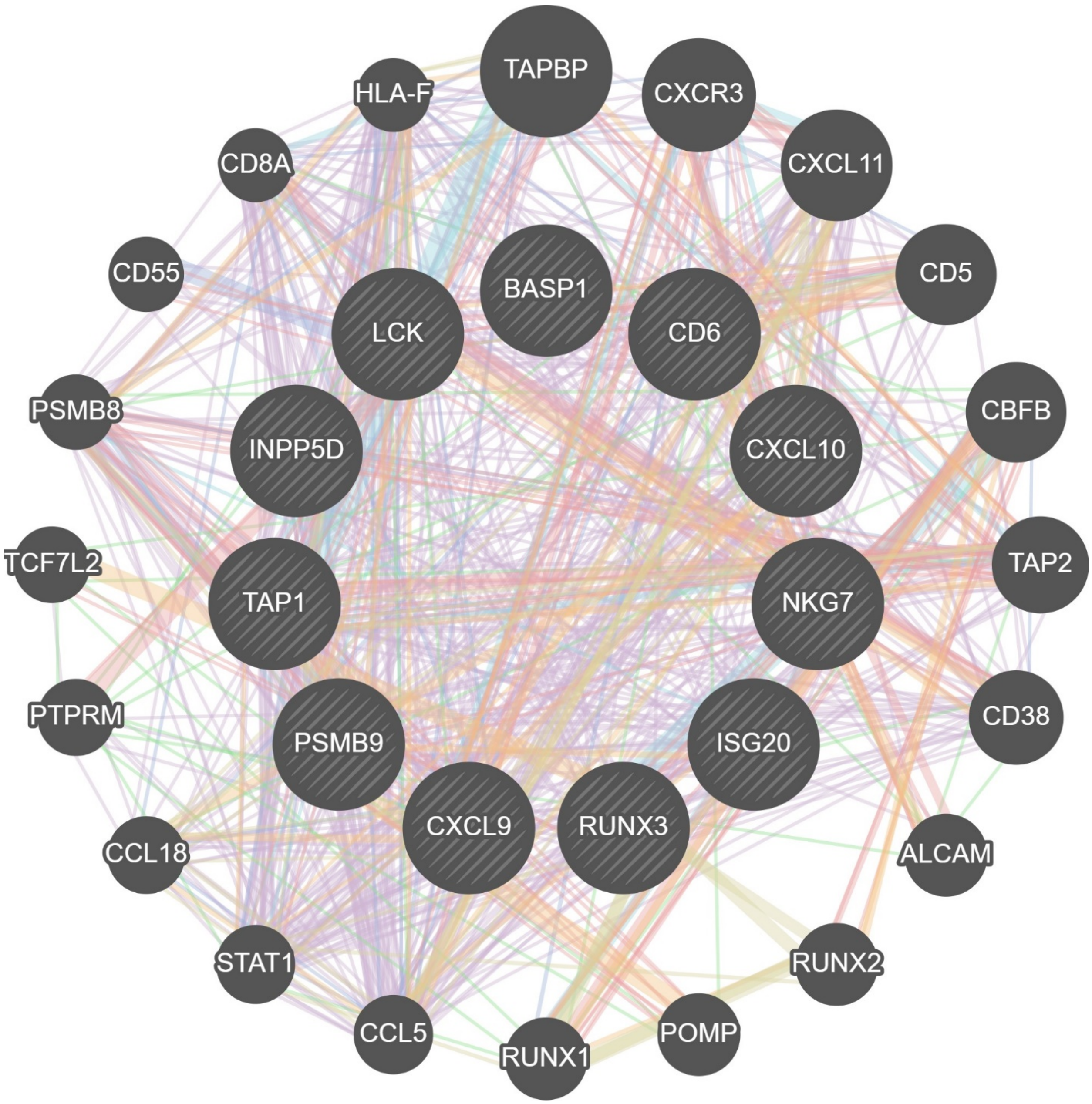

Supplement: S1 Fig — A network view of the CRM genes. Cross-hatched bubbles are the CRM genes while solid bubbles indicate partners identified through network analysis. Connections are color-coded based on the type of interaction. Red, physical interaction; purple, co-expression; orange, predicted; blue, co-localization; turquoise, pathway; green, genetic interactions; yellow, shared protein domains. (PDF) [file pone.0220052.s001.pdf]
