# Supplementary material for: A urinary Common Rejection Module (uCRM) score for non-invasive kidney transplant monitoring
Source: PLoS One. 2019 Jul 31;14(7):e0220052. doi: 10.1371/journal.pone.0220052 (PMC6668802; doi:10.1371/journal.pone.0220052)

**Supplemental Figure S2. uCRM score classification performance inclusive of BKVN phenotype.** A. ROC curve of the uCRM score in discriminating between AR and the combination of bAR and STA phenotypes (AUC = 0.9677, P < 0.0001). B. Scatter dot plot of the uCRM score for AR, bAR, STA, and BKVN phenotypes. Significance was determined by nonparametric Kruskal-Wallis test with Dunn's multiple comparisons correction. C. ROC curve of the uCRM score in discriminating between AR and the combination of bAR and STA phenotypes (AUC = 0.9111, P < 0.0001). * < 0.05. ** < 0.01. *** P < 0.001. **** P < 0.0001.

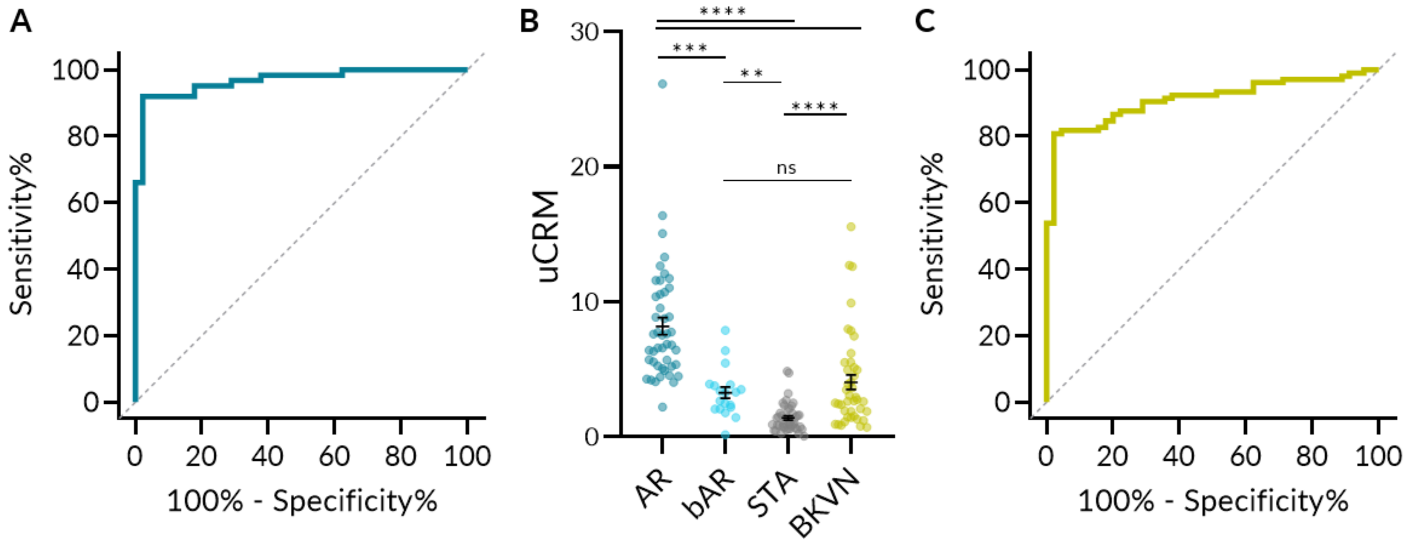

Supplement: S2 Fig — A. ROC curve of the uCRM score in discriminating between AR and the combination of bAR and STA phenotypes (AUC = 0.9677, P < 0.0001). B. Scatter dot plot of the uCRM score for AR, bAR, STA, and BKVN phenotypes. Significance was determined by nonparametric Kruskal-Wallis test with Dunn’s multiple comparisons correction. C. ROC curve of the uCRM score in discriminating between AR and the combination of bAR and STA phenotypes (AUC = 0.9111, P < 0.0001). * < 0.05. ** < 0.01. *** P < 0.001. **** P < 0.0001. (PDF) [file pone.0220052.s002.pdf]
