# Supplementary material for: A urinary Common Rejection Module (uCRM) score for non-invasive kidney transplant monitoring
Source: PLoS One. 2019 Jul 31;14(7):e0220052. doi: 10.1371/journal.pone.0220052 (PMC6668802; doi:10.1371/journal.pone.0220052)

Supplemental Figure S3. Lack of Association of uCRM Score with Additional Histological Parameters. The correlation between the uCRM score and various histological parameters was determined. Other than the tubulitis (t) and interstitial inflammation (ii) scores that were significantly correlated (**Figure 5A and 5B**), none of tubular atrophy (ta), glomerulosclerosis (gs), mesangial matrix (mm), intimal proliferation (cv), medial arteriolar hyaline (ah), tubular vacuolization (tv), arteritis (v), ora cute glomerulitis (g) scores were significantly correlated.

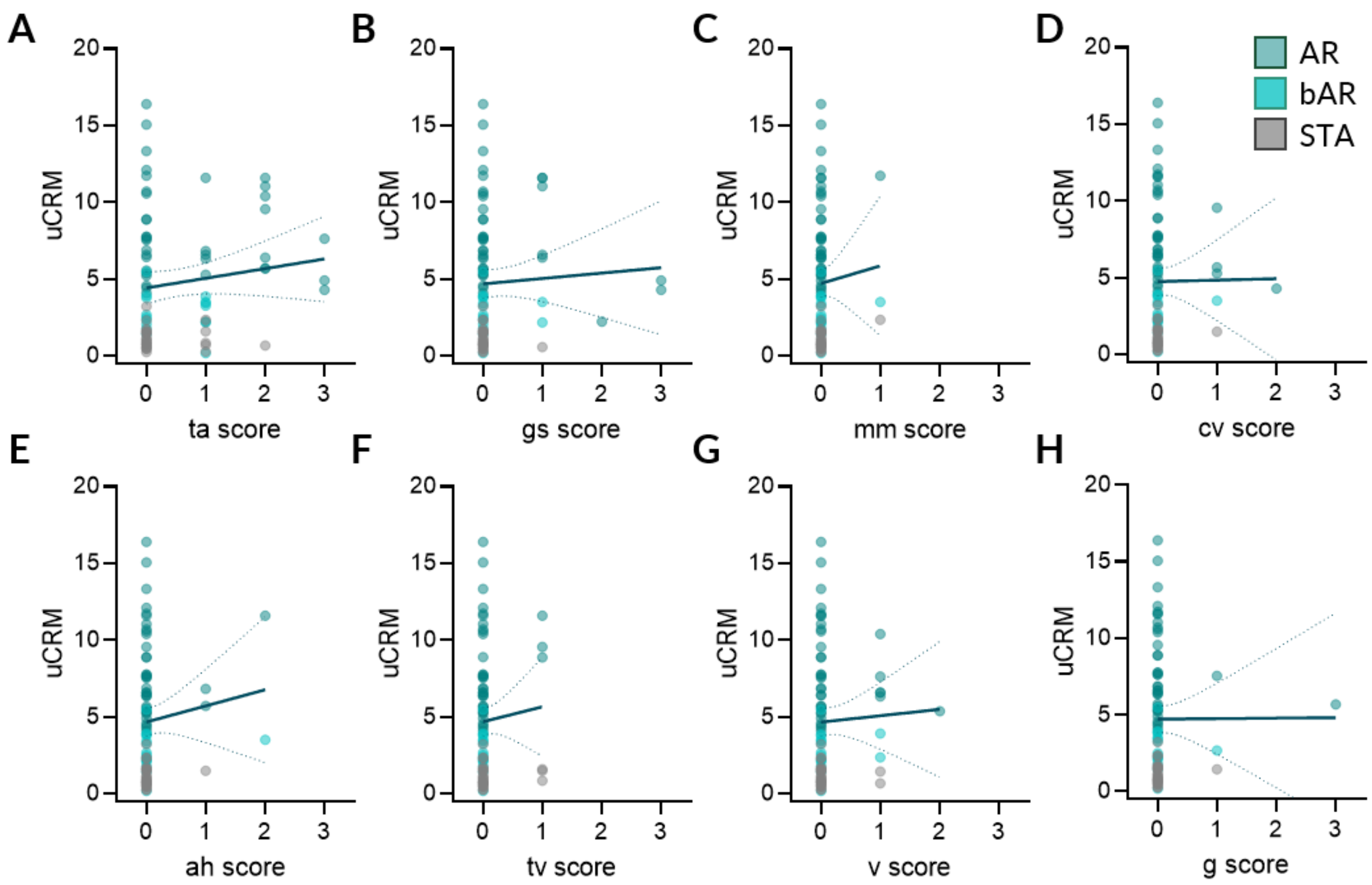

Supplement: S3 Fig — The correlation between the uCRM score and various histological parameters was determined. Other than the tubulitis (t) and interstitial inflammation (ii) scores that were significantly correlated (Fig 5A and 5B), none of tubular atrophy (ta), glomerulosclerosis (gs), mesangial matrix (mm), intimal proliferation (cv), medial arteriolar hyaline (ah), tubular vacuolization (tv), arteritis (v), ora cute glomerulitis (g) scores were significantly correlated. (PDF) [file pone.0220052.s003.pdf]
