# Supplementary material for: A urinary Common Rejection Module (uCRM) score for non-invasive kidney transplant monitoring
Source: PLoS One. 2019 Jul 31;14(7):e0220052. doi: 10.1371/journal.pone.0220052 (PMC6668802; doi:10.1371/journal.pone.0220052)
Supplement: S1 Table — (PDF) [file pone.0220052.s004.pdf]

Supplemental Table S1: Primer information for primers used for uCRM assay

Vendor	Catalog no.	Item no.	Gene Symbol
Thermo Fisher Scientific	4331182	Hs00234720_g1	BASP1
Thermo Fisher Scientific	4331182	Hs00171042_m1	CXCL10
Thermo Fisher Scientific	4331182	Hs00171065_m1	CXCL9
Thermo Fisher Scientific	4331182	Hs00183290_m1	INPP5D
Thermo Fisher Scientific	4331182	Hs00158122_m1	ISG20
Thermo Fisher Scientific	4331182	Hs00178427_m1	LCK
Thermo Fisher Scientific	4331182	Hs01120688_g1	NKG7
Thermo Fisher Scientific	4331182	Hs00544762_m1	PSMB9
Thermo Fisher Scientific	4331182	Hs00231709_m1	RUNX3
Thermo Fisher Scientific	4331182	Hs00388675_m1	TAP1
Thermo Fisher Scientific	4331182	Hs00198752_m1	CD6
Thermo Fisher Scientific	4331182	Hs03003631_g1	18S[A]

[A]Reference gene
